# Rapid Detection, Complete Genome Sequencing, and Phylogenetic Analysis of Porcine Deltacoronavirus

**DOI:** 10.3201/eid2008.140526

**Published:** 2014-08

**Authors:** Douglas Marthaler, Lindsey Raymond, Yin Jiang, James Collins, Kurt Rossow, Albert Rovira

**Affiliations:** University of Minnesota Veterinary Diagnostic Laboratory, Saint Paul, Minnesota, USA

**Keywords:** Porcine deltacoronavirus, rRT-PCR, phylogenetic analysis, United States, viruses

## Abstract

In February 2014, porcine deltacoronavirus (PDCoV) was identified in the United States. We developed a PDCoV real-time reverse transcription PCR that identified PDCoV in 30% of samples tested. Four additional PDCoV genomes from the United States were sequenced; these had ≈99%–100% nt similarity to the other US PDCoV strains.

Coronaviruses belonging to the *Coronavirinae* subfamily are divided into 3 genera, *Alphacoronavirus*, *Betacoronavirus*, and *Gammacoronavirus* (*1*). Woo et al. investigated the presence of coronaviruses in birds and mammals from Hong Kong and identified a new *Coronavirinae* genus, *Deltacoronavirus* (*2**,**3*). Of 169 swine samples tested, 10% were positive for porcine deltacoronavirus (PDCoV), and 2 complete PDCoV genomes were generated and analyzed (*3*). 

On February 11, 2014, the Ohio Department of Agriculture officially announced the identification of PDCoV in the United States. Furthermore, the University of Minnesota Veterinary Diagnostic Laboratory (Saint Paul, MN, USA) and Iowa State University Veterinary Diagnostic Laboratory (Ames, IA, USA) sequenced a US PDCoV strain, which had an ≈99% nt identity to the 2 China PDCoV strains. In addition, the Ohio Department of Agriculture released 2 more complete PDCoV genomes from the United States (GenBank accession nos. KJ569769 and KJ462462), for a total of 4 complete PDCoV genomes (*4*,*5*). We designed a real-time reverse transcription PCR (rRT-PCR) to rapidly identify PDCoV, and 4 additional PDCoV strains were sequenced to further characterize PDCoV in the United States.

## The Study

During January 6–February 27, 2014, we tested a total of 293 porcine samples—90 fecal swab samples, 75 fecal samples, 54 saliva samples, 52 intestinal homogenate samples, 2 vomit samples, 19 feed samples, and 1 environmental sample—from Ohio (108 samples), Michigan (63), Illinois (38), Minnesota (24), Nebraska (25), South Dakota (24), Missouri (3), and Canada (8) with the new PDCoV rRT-PCR (Table 1). The PDCoV rRT-PCR design, comparison, sensitivity, and specificity are described in the Technical Appendix. We selected samples for PDCoV testing in accordance with veterinarians’ requests to investigate the presence of PDCoV in pigs with diarrhea. The samples were homogenized and the RNA extracted by using previously described methods (*6*,*7*). Of 293 porcine samples tested, 89 (30%) were PDCoV positive; we did not detect transmissible gastroenteritis virus (TGEV) in any samples tested (Table 1). Of the 89 PDCoV-positive samples, 20 samples (22% [11 fecal, 6 intestinal homogenate, 2 fecal swab, and 1 feed]) were negative for TGEV, porcine epidemic diarrhea virus (PEDV), rotavirus A (RVA), rotavirus B (RVB), and rotavirus C (RVC); 69 (78%) PDCoV-positive samples were positive for PEDV, RVA, RVB, or RVC. Co-infection with PDCoV and RVC were most common (52 [58%] samples). Although most (27 [30%]) PDCoV co-infections were with only 1 viral pathogen, 15 (17%) were positive for PEDV, RVA, RVB, and RVC.

**Table 1:** Characteristics and results of samples tested for PDCoV, United States, January 6–February 27, 2014*

Characteristic	Positive samples, no. (%)
Sample type tested	
Fecal swab, n = 90	15 (17)
Feces, n = 75	30 (40)
Saliva, n = 54	10 (19)
Intestines, n = 52	27 (52)
Feed, n = 19	6 (32)
Vomit, n = 2	1 (50)
Environment, n = 1	0
Total, n = 293	89 (30)
Location	
Ohio, n = 108	41 (38)
Illinois, n = 38	27 (71)
Minnesota, n = 24	7 (29)
Nebraska, n = 25	14 (56)
Michigan, n = 63	0
South Dakota, n = 24	0
Canada, n = 8	0
Missouri, n = 3	0
Total, n = 293	89 (30)
rRT-PCR results for PDCoV-positive samples	
Total, n = 89	
PEDV	29 (33)
RVA	35 (39)
RVB	33 (37)
RVC	52 (58)
PDCoV only	20 (22)
PDCoV + any co-infections	69 (78)
PDCoV + 1 pathogen	
PEDV	5 (19)
RVA	6 (22)
RVB	4 (15)
RVC	12 (44)
Total	27 (30)
PDCoV + 2 pathogens	
PEDV + RVA	0
PEDV + RVB	1 (5)
PEDV + RVC	3 (16)
RVA + RVB	0
RVA + RVC	9 (47)
RVB + RVC	6 (32)
Total	19 (21)
PDCoV + 3 pathogens	
PEDV + RVA + RVB	1 (13)
PEDV + RVA + RVC	1 (13)
PEDV + RVB + RVC	3 (38)
RVA + RVB + RVC	3 (38)
Total	8 (9)
PDCoV + 4 pathogens	
PEDV + RVA + RVB + RVC	15 (17)

*PDCoV, porcine deltacoronavirus; rRT-PCR, real-time reverse transcription PCR; PEDV, porcine epidemic diarrhea virus; RVA,,rotavirus A; RVB, rotavirus B; RVC, rotavirus C.

We detected PDCoV in 52% of intestinal samples and 40% of fecal samples; 32% of feed samples and 19% of saliva samples tested positive for PDCoV. Of the 8 different locations tested for PDCoV, samples from Ohio (41), Illinois (27), Minnesota (17), and Nebraska (14) were positive for PDCoV; samples from Michigan, South Dakota, Missouri, and Canada were negative for PDCoV (Table 1).

From the PDCoV-positive samples, we selected 4 for complete genome sequencing (Technical Appendix). The 8 US PDCoV complete genome sequences were 99.9%–100% nt identical to each other and 98.9%–100% nt identical to the China PDCoV strains (Table 2). The envelope and membrane gene segments were the most conserved and had a 100% nt identity, and the nonstructural (NS) 6 accessory gene had the lowest nucleotide identity (98.9%–100%) within the US strains. Compared with segments of the China strains, the envelope gene segment was the most conserved (99.6% nt identity), and the spike gene segment was the most diverse (98.5%–98.8% nt identity). The China PDCoV strain HKU15-155 contained two 3-nt deletions in the spike gene and 3′ untranslated region; the China strain HKU15–44 and US strains lacked this deletion. Inversely, PDCoV China strain HKU15–44 contained a nucleotide deletion in the 3′ untranslated region that was not present in the US PDCoV strains.

**Table 2:** Nucleotide identities of porcine deltacoronavirus strains*

Strain	Nucleotide identity, %							
	Genome	ORF1	Spike	Envelope	Membrane	Nucleocapsid	NS6	NS7
HKU strains	99.1	99.1	98.8	100	99.4	99.6	98.9	99.3
HKU vs. US	98.9–99.2	98.9–99.4	98.5–98.8	99.2–99.6	98.9–99.2	98.8–99	98.9–100	98.8–99.0
US	99.9–100	99.9–100	99.7–100	99.6–100	99.8–100	99.4–100	99.3–100	99.2–100
*ORF, open reading frame; NS, nonstructural.								

In the complete genome phylogenetic tree, the 8 US PDCoV strains clustered with China PDCoV strain HKU15-155 instead of HKU15-44 (Figure, panel A). With the open reading frame 1, spike, envelope, membrane, and nucleocapsid gene segments and NS7 accessory gene phylogenetic trees, the US PDCoV strains clustered separately from the China PDCoV strains (Figure, panel B). The phylogenetic tree for the NS6 accessory gene had a different clustering pattern from the China strains (Figure, panel C). China PDCoV strain HKU15-44 clustered with Illinois133 and Illinois134, and China PDCoV strain HKU15–155 clustered independently.

**Figure:**
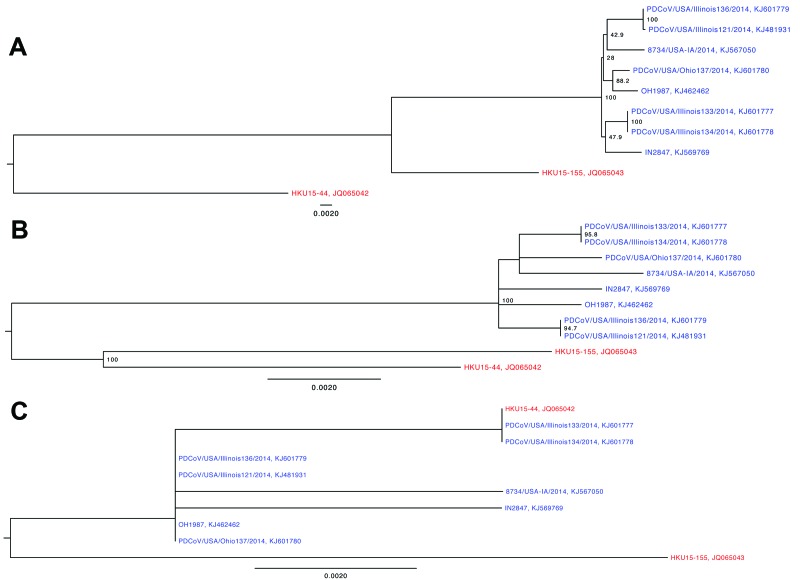
Phylogenetic trees of the complete porcine deltacoronavirus (PDCoV) genome (A), spike gene (B), and nonstructural protein 6 (NS6) accessory gene (C). US strains are in blue; China strains are in red. Bootstrap values >70% are illustrated. Scale bar indicates nucleotide substitutions per site.

## Conclusions

The PDCoV rRT-PCR is a fast and accurate detection method that can be used to diagnose PDCoV infection. Identification of PDCoV in 30% of samples tested indicates that PDCoV is a common viral pathogen of pigs in the midwestern United States. We identified positive PDCoV in 20 (22%) samples that were negative for TGEV, PEDV, RVA, RVB, and RVC, but PDCoV co-infections were more common (69 [78%] samples), especially with RVC (52 [58%]). Although the samples from Canada were negative for PDCoV, the Animal Health Laboratory has confirmed that 6 Ontario farms contain PDCoV. Because we selected samples on the basis of clinical diarrhea and geographic location was limited, the results do not accurately reflect the prevalence of PDCoV in North America. In addition, the presence of PDCoV RNA in feed does not indicate infectivity of the virus. The prevalence of PDCoV in North America is unknown, and the new PDCoV rRT-PCR can be used to access the prevalence in the United States and Canada.

Phylogenetic analysis of the US PDCoV strains indicates a common ancestor with the China PDCoV strains. The China PDCoV strains are the only available sequences, and we cannot state that the US PDCoV strains originated in China. Because little is known about PDCoV, the US PDCoV parental strain may never be discovered. The NS6 phylogenetic tree branched differently from the other PDCoV gene segments; therefore, the NS6 accessory gene may evolve differently from the other gene segments. Complete genomes of PDCoV from other countries are needed to increase understanding of the origin, phylogenetic relationship, and evolution of the US PDCoV strains.

The date that PDCoV was introduced into the United States is unknown. Because the sequenced PDCoV samples were from a similar geographic location, the 99.9%–100% nt identity does not correlate with the possible genetic diversity within the United States. An alternate hypothesis would indicate that PDCoV has been an undiagnosed pathogen of pigs in the United States and, like RVB, has been circulating there for an extended period (*6*) or that PDCoV might be a secondary infection to other enteric pathogens. PDCoV pathogenesis and retrospective surveillance studies are needed to answer these epidemiologic questions in the United States and to determine PDCoV prevalence worldwide.

In conclusion, a PDCoV rRT-PCR was designed to accurately detect PDCoV in a variety of samples. Complete genome analysis of the US PDCoV strains showed that they share 99.9%–100% nt identity and a common ancestor with the only available PDCoV sequences, the China PDCoV strains.

Technical Appendix Real-time reverse transcription PCR, complete genome sequencing and phylogenetic analysis of porcine deltacoronavirus.
