# Supplementary material for: Rapid Detection, Complete Genome Sequencing, and Phylogenetic Analysis of Porcine Deltacoronavirus
Source: Emerg Infect Dis. 2014 Aug;20(8):1347–50. doi: 10.3201/eid2008.140526 (PMC4111195; doi:10.3201/eid2008.140526)
Supplement: Technical Appendix — Real-time reverse transcription PCR, complete genome sequencing and phylogenetic analysis of porcine deltacoronavirus. [file 14-0526-Techapp-s1.pdf]

Article DOI: http://dx.doi.org/10.3201/eid2008.14-0526

Rapid Detection, Complete Genome Sequencing, and Phylogenetic Analysis of Porcine Deltacoronavirus

Technical Appendix

Real-Time Reverse Transcription PCR

Initially, 3 separate porcine deltacoronavirus (PDCoV) real-time reverse transcription PCR (rRT-PCR) were designed from 2 nucleotide sequences available from GenBank (accession nos. JQ065042 and JQ065043), targeting the open reading frame 1 gene segment (forward primer, CAGTACCATCAAAAGATGCTGAAATC; reverse primer, ACCATAGAATTTGGTTGTTCCAATC; and probe, TTCACTTGCACGCAATCAGACCATCG), the spike gene (forward primer, ATCTGCGCACTGATACCTGTAGTT; reverse primer, TCATCTCACAAGCACCAGATTCA; and probe, CCTGTCAGCAGTAAACAATGGCATGTCATT); and the membrane gene segment (forward primer, ATCGACCACATGGCTCCAA; reverse primer, CAGCTCTTGCCCATGTAGCTT; and probe, FAM-CACACCAGTCGTTAAGCATGGCAAGCT-BHQ). The PDCoV primer and hydrolysis probe sets used with the Ambion Path-ID Multiplex One-Step RT-PCR Kit (Life Technologies, Austin, TX, USA) and 5 µL of RNA with the 7500 Fast Real-Time PCR System (Life Technologies) on the Fast Mode setting and the following thermal cycling conditions: reverse transcription, 10 min at 48°C; Taq activation, 10 min at 95°C; followed by 40 cycles of 15 sec at 95°C and 45 sec at 60°°C.

The 3 PDCoV RT-PqCRs were tested with 240 porcine samples. The open reading frame 1 PDCoV rRT-PCR was not tested on the remaining 53 porcine samples because of the reduced sensitivity of the assay compared with the membrane and spike rRT-PCR (data not shown). A gBlock Gene Fragment (IDT, Coralville, IA, USA) was ordered to estimate the detection limit of the membrane and spike PDCoV rRT-PCRs, which both had a detection limit of 2 PDCoV

copies per reaction. Although the paired *t* test did not indicate significant difference between the percentage of detection of the membrane and spike rRT-PCRs (n = 293, p = 0.72), the membrane PDCoV rRT-PCR yielded slightly lower cycle threshold and was chosen as the PDCoV rRT-PCR for the University of Minnesota Veterinary Diagnostic Laboratory. The membrane PDCoV rRT-PCR did not detect 60 common swine pathogens, transmissible gastroenteritis virus, porcine respiratory coronavirus, porcine hemagglutinating encephalomyelitis virus, porcine cytomegalovirus, North American porcine reproductive and respiratory syndrome virus, European porcine reproductive and respiratory syndrome virus, pseudorabies virus, swine influenza virus H1, swine influenza virus H2, swine influenza virus H3, encephalomyocarditis virus, porcine enterovirus, classical swine fever, porcine hepatitis E virus, porcine parvovirus type I, porcine adenovirus, porcine circovirus type I, porcine circovirus type II, porcine picornavirus, porcine lymphotropic gamma herpes virus 1, porcine lymphotropic gamma herpes virus 2, porcine hokovirus, beta-hemolytic *Escherichia coli*, non–beta-hemolytic *Escherichia coli, Pasteurella multocida*, *Salmonella cholerasuis, Actinobacillus pleuropneumoniae, Bordetella bronchiseptica*, *Clostridium perfringens* type A, *Clostridium perfringens* type C, *Salmonella* Typhimurium, *Brachyspira hyodysenteriae*, *Brachyspira pilosicoli, Brachyspira murdochii*, *Brachyspira intermedia*, *Brachyspira innocens, Actinobacillus suis, Actinobacillus rossi*, *Actinobacillus minor*, *Actinobacillus indolicus*, *Actinobacillus equuli, Actinobacillus pyogenes*, *Streptococcus suis, Enterococcus durans*, *Yersinia enterocolitica*, *Campylobacter coli, Campylobacter jejuni*, *Staphylococcus aureus*, *Mycoplasma hyorhinis*, *Mycoplasma hyopneumoniae*, *Mycoplasma hyosynoviae*, *Haemophilus parasuis*, *Leptospira* species, and *Erysipelothrix rhusiopathiae*.

Complete Genome Sequencing and Phylogenetic Analysis

Four positive PDCoV samples were selected for next-generation sequencing by clients' requests as previously described (*1*,*2*). The 4 new and 6 previous PDCoV genomes were aligned with Mauve in Geneious Pro (*3*) (http://www.geneious.com/). Phylogenetic trees were constructed by using the maximum-likelihood method with a nucleotide substitution model of general-time reversible with a γ-distributed among-site rate variation and mid-rooted and bootstrapped with 1,000 iterations variation (*4*).

References

1. Marthaler D, Jiang Y, Otterson T, Goyal S, Rossow K, Collins J. Complete genome sequence of porcine epidemic diarrhea virus strain USA/Colorado/2013 from the United States. Genome Announc. 2013;1:e00555–13. PubMed http://dx.doi.org/10.1128/genomeA.00555-13

2. Marthaler D, Jiang Y, Collins J, Rossow K. Complete genome sequence of strain SDCV/USA/Illinois121/2014, a porcine deltacoronavirus from the united states. Genome Announc. 2014;2:e00218–14. PubMed http://dx.doi.org/10.1128/genomeA.00218-14

3. Darling AC, Mau B, Blattner FR, Perna NT. Mauve: multiple alignment of conserved genomic sequence with rearrangements. Genome Res. 2004;14:1394–403. PubMed http://dx.doi.org/10.1101/gr.2289704

4. Guindon S, Gascuel O. A simple, fast, and accurate algorithm to estimate large phylogenies by maximum likelihood. Syst Biol. 2003;52:696–704. PubMed http://dx.doi.org/10.1080/10635150390235520
